# *K-ras* Mutational Status in Cytohistological Tissue as a Molecular Marker for the Diagnosis of Pancreatic Cancer: A Systematic Review and Meta-Analysis

**DOI:** 10.1155/2014/573783

**Published:** 2014-07-16

**Authors:** Jing Yang, Jingjing Li, Rong Zhu, Huawei Zhang, Yuanyuan Zheng, Weiqi Dai, Fan Wang, Miao Shen, Kan Chen, Ping Cheng, Yan Zhang, Chengfen Wang, Junshan Wang, Yujing Xia, Jie Lu, Yingqun Zhou, Chuanyong Guo

**Affiliations:** Department of Gastroenterology, Shanghai Tenth People's Hospital, Tongji University School of Medicine, Shanghai 200072, China

## Abstract

*Background*. More clinically meaningful diagnostic tests are needed in pancreatic cancer (PC).* K-ras* mutations are the most frequently acquired genetic alteration. *Methods*. Original research articles involving the diagnostic accuracy of *K-ras* mutation detection in PC were selected. Data were presented as forest plots and summary receiver operating characteristic (SROC) curve analysis was used to summarize the overall test performance. *Results*. We assessed 19 studies from 16 published articles. The reports were divided into three groups according to the process used to obtain the test material. The summary estimates for detecting *K-ras* status using an invasive method (fine needle aspiration (FNA), endoscopic retrograde cholangiopancreatography (ERCP), or surgery) were better than cytology: the pooled sensitivity was 77% (95% confidence interval (CI): 74–80%) versus 54% (95% CI: 47–61%); specificity was 88% (95% CI: 85–91%) versus 91% (95% CI: 83–96%); and diagnostic odds ratio (DOR) was 20.26 (11.40–36.03) versus 7.52 (95% CI: 2.80–20.18), respectively. When two procedures were combined, the diagnostic accuracy was markedly improved. *Conclusions*. The analysis of *K-ras* mutations in pancreatic tissue has a promising diagnostic significance in PC. Further valuable studies are needed.

## 1. Introduction

Pancreatic cancer (PC) is one of the most aggressive malignancies, with an overall 5-year survival rate of only 3% [1]. The reasons for this poor prognosis are due to its early dissemination, its lack of early specific symptoms, and its late diagnosis. At the time of diagnosis, patients with PC usually have locally advanced disease and even metastases, precluding curative surgery, which is currently the only possible curative method for PC in the very early stage [2]. Early detection of PC is therefore extremely important to improve patient survival. Imaging methods are not accurate enough to detect early lesions, assess tumor stage or resectability, and even discriminate between benign and malignant lesions [3]. Currently, CA 19-9 is considered the best-validated serum marker in patients with PC, but it has limited use in the early diagnosis of PC and in monitoring progression of the disease. More clinically meaningful and efficient diagnostic strategies such as molecular markers capable of identifying PC during the curative stage of the disease are urgently needed.

The* K-ras* gene encodes a protein that acts as a mediator in intracellular processes and has an important role in cell proliferation and differentiation [4]. Present in 75%–90% of tumors, activating point mutations in the* K-ras* oncogene is the most frequently acquired genetic alteration in PC [5, 6], the most common of which is substitution of the wild-type glycine residue (codon 12) by cysteine, arginine, valine, or aspartic acid.* K-ras* mutations can be detected—with substantially different accuracy—in pancreatic tissue, pancreatic juice, bile, plasma, and stools.* K-ras* tests may thus have different diagnostic properties. In plasma or stools,* K-ras* mutations are detected at a much lower frequency than in cytohistological materials (pancreatic juice, pancreatic duct brushings, duodenal aspirates, or tissue) [7–9]; therefore, we mainly identified studies that evaluated the diagnostic accuracy using these methods. In fact, the detection of* K-ras* mutation could not only be useful in the diagnosis, but also in staging, prognosis, evaluation of response to therapy, and follow-up of PC.

Numerous studies have been carried out to determine the diagnostic utility of* K-ras* mutation detection; however, results are heterogeneous and conflicting. The objective of the present review was to analyze the results from a systematic selection of research papers that evaluated the utility of detecting* K-ras* mutational status in different cytohistological materials for the diagnosis of PC using an invasive method.

## 2. Methods

### 2.1. Inclusion and Exclusion Criteria

Articles were preselected based on the following criteria. Original research articles that evaluated the diagnostic accuracy of* K-ras* mutation detection for the diagnosis of PC in at least 30 patients were included. Studies that evaluated the diagnostic validity of* K-ras* in combination with other diagnostic procedures were included. The method used to retrieve samples for DNA extraction and* K-ras* determination was limited to invasive techniques (FNA, ERCP, or surgery). Studies which did not provide exact raw data on sensitivity or specificity were excluded. Abstracts, letters, editorials, expert opinions, reviews without original data, case reports, and studies lacking control groups were also excluded. No restriction was set on the study design, year of publication, or publication status.

### 2.2. Identification of Studies

A comprehensive systematic literature review of original research assessing the diagnostic accuracy of* K-ras* was performed by searching the following electronic databases up to March 2014: PubMed/Medline, Embase, Cochrane Database of Systematic Reviews, Cochrane Central Register of Controlled Trials, Science Citation Index (ISI Web of Science), Chinese Biomedical Literature Database (CBM), and Chinese National Knowledge Infrastructure (CNKI) [10, 11]. In addition, references from the included articles and relevant published reports were hand searched. Subject headings and keywords used in the search process were as follows: (1)* K-ras*,* kirsten-ras*, and* ki-ras*; (2) PC, pancreatic neoplasms, pancreatic cancer, adenocarcinomas of the pancreas, and pancreatic adenocarcinomas. We did not use keywords or indexing terms for diagnostic accuracy to avoid missing relevant studies.

### 2.3. Study Selection

All the studies were reviewed by two reviewers (Jing Yang and Jingjing Li) independently based on titles and abstracts, and then the full texts of potentially eligible studies were retrieved for further assessment. Disagreements between the reviewers were resolved by consensus. The authors were contacted for further study details when necessary.

### 2.4. Data Extraction

Using a structured form, the following data were extracted from the included studies by two reviewers (Jing Yang and Jingjing Li) independently: author, year of publication, journal, study design, number of patients initially included in the study, number of patients that contributed to the evaluation of* K-ras*, assay type for markers, and raw data on analysis of sensitivity and specificity (the number of true positive, false negative, true negative, and false positive results) to compare patients diagnosed with PC versus all other patients included and to determine whether the study evaluated the diagnostic accuracy of another marker in addition to* K-ras* mutation. According to the sample type and the method used to obtain the material, we divided the studies into three groups: minimally invasive (FNA), moderately invasive (ERCP), and very invasive (surgery).

When the articles described two different types of samples or presented two separate patient populations, the text referring to the clinical application of each setting was extracted, and the results were recorded as two separate studies. Overall, the agreement between the two reviewers for data extraction was 87.5%. Discrepancies were resolved by consultation with the third reviewer (Rong Zhu).

### 2.5. Assessment of Methodological Quality

The quality of each study was assessed according to the adapted quality criteria from the QUADAS-2 checklist recommended by the Cochrane Collaboration [12, 13]. The QUADAS-2 tool comprises four domains: patient selection, index test, reference standard, and flow and timing. Each domain is assessed in terms of risk of bias, and the first three domains are also assessed in terms of concerns regarding applicability. Signaling questions are included to help judge risk of bias. This revised checklist allows for more transparent rating of bias and applicability of primary diagnostic accuracy studies.

### 2.6. Representative Patient Spectrum

Patients with upper abdominal pain, development or worsening of diabetes mellitus, elevated serum pancreatic enzymes and/or tumor markers, and abnormal imaging findings including the dilated pancreatic/biliary duct and suspicious pancreatic masses were selected as being at high risk for PC. It is in these target populations that molecular markers are most urgently needed.

Histopathology is the currently accepted reference standard recommended for PC. If histopathology is not available, PC diagnosis is usually established by various imaging methods (such as ERCP, US, MRI, CT, and EUS), and a compatible clinical course (when death occurred within the first year after diagnosis, with clinical evolution compatible with disseminated cancer disease). Other types of neoplasia were diagnosed on the basis of pathological findings. The diagnosis of chronic pancreatitis was usually based on standard clinical criteria and ERCP findings.

### 2.7. Data Analysis

Using MetaDisc (version 1.4; Clinical Biostatistics Unit, Ramon y Cajal Hospital, Madrid, Spain), the Spearman correlation coefficient was calculated to estimate if there was a threshold effect. The overall sensitivity, specificity, and diagnostic odds ratio (DOR) were then calculated. Data were presented as forest plots with RevMan5.2 (The Nordic Cochrane Centre, The Cochrane Collaboration, 2012), which display the results of individual studies with the corresponding 95% confidence intervals (CIs). SROC curve analysis was used to summarize the overall test performance. Midas model for Stata (version 12.0; StataCorp, College Station, TX, USA) was used to construct the funnel plots and calculate *P* values. Publication bias existed when a *P* value <0.05 was observed. Meta-regression was also performed in an attempt to explain the observed heterogeneity and to explore how certain methodological characteristics influenced the observed sensitivity and specificity. Comparisons between subgroups were made using this method.

## 3. Results

**Figure 1:**
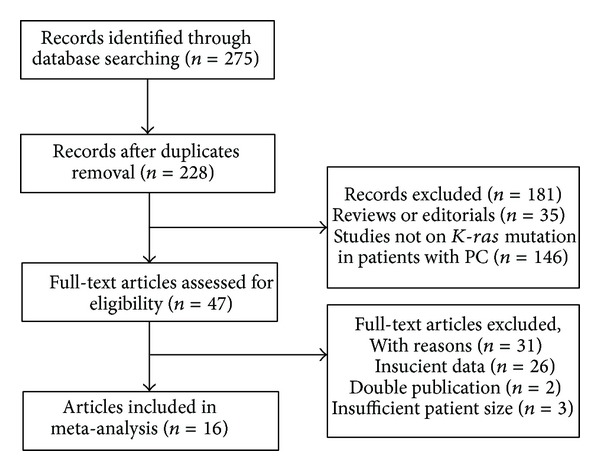
Flow diagram showing the process of article selection for the meta-analysis.

We searched suitable articles published from 1985 through 2014 (30 years), and a total of 275 articles were found, of which 47 publications were considered to be eligible for inclusion in the analysis. After full-text review, 31 articles were excluded: 26 articles were excluded because they did not allow the calculation of sensitivity or specificity; two were excluded because of a suspected overlap study population or a duplicate publication; and three were excluded because of insufficient patient size. In total, 19 studies from 16 published articles were included in our meta-analysis, including three studies which detected* K-ras* mutations in serum [14–29].  Figure 1 shows a flow diagram describing the search and selection process.

Five studies presented results for more than one type of sample (e.g., needle biopsy, pancreatic juice, and even serum) or for more than one patient subgroup (e.g., according to the presence or absence of a pancreatic mass). Therefore, of 16 articles, we present the results from a total of 19 independent patient series, herein referred to as individual studies. Five studies obtain the samples for* K-ras* detection from surgery, ten from ERCP and the remaining four from FNA.

18 of the 19 studies (94.7%) detected* K-ras* mutations in codon 12, and nine studies (52.6%) detected* K-ras* mutations using restriction fragment length polymorphism (RFLP)/PCR. In 12 studies, the ethnicity was Asian (Chinese or Japanese). Five of 16 articles (31.25%) reported the tumour stage of the pancreatic cancer patients included. A summary of the analytical details of* K-ras* mutational testing is shown in Table 1. The number of patients in each of the 19 studies differed significantly, which may be a source of heterogeneity.

### 3.1. Quality of the Studies Identified

**Figure 2:**
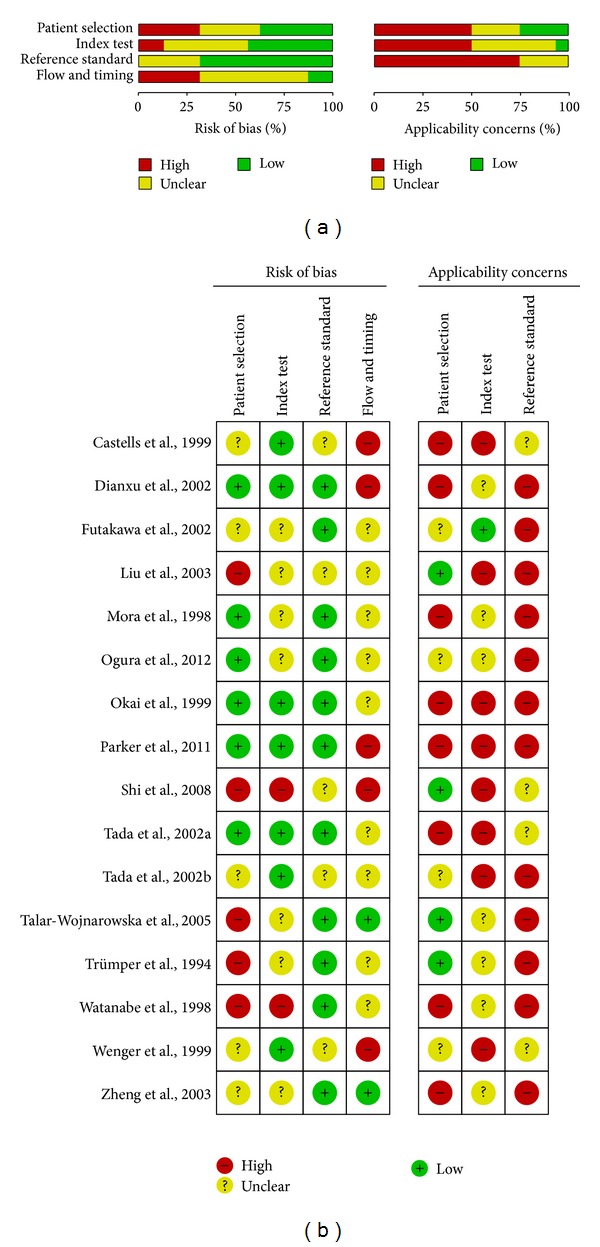
Summary of the methodological quality of included studies on the basis of review authors' judgments on the four domains of the QUADAS-2 checklist for each study.

QUADAS-2 criteria were used to evaluate the quality of all the 19 studies from 16 published articles (Figure 2). However, the quality was unsatisfactory. Only four studies used a perspective design. Notably, the selection criteria were not clearly described in nine articles (56.25%), and various studies did not report sociodemographic or clinical details of the final patient population. Less than half of the studies (seven, 43.75%) stated that the* K-ras* mutational status of patients was interpreted blinded to the definitive diagnosis. Both situations may lead to reviewer bias and inflated estimates of diagnostic accuracy. One study recruited healthy subjects in the control group. All the studies reported the diagnostic standard of PC. Of 16 studies that did not report* K-ras* results in all patients who entered the study, four (25%) failed to explain why this had occurred. Of the 12 that did explain, common reasons were failure of DNA amplification and problems with sample collection.

### 3.2. Summary Diagnostic Accuracy of* K-ras* Mutation Using an Invasive Method (FNA, ERCP, and Surgery) for PC

**Figure 3:**
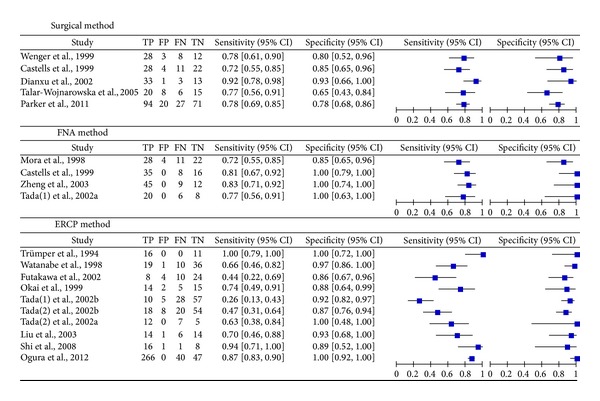
Forest plot of the meta-analysis of each index.

The DerSimonian-Laird (random effects) model was used to calculate the pooled value. The sensitivity of* K-ras* mutation levels in these studies ranged from 72% to 83%, 26% to 100%, and 72% to 92% using FNA, ERCP and surgical tissues in the diagnosis of PC, respectively, while the specificity ranged from 85% to 100%, 86% to 100%, and 65% to 93%, respectively. We included the paired sensitivity and specificity with 95% CIs for each study in the forest plot, and significant heterogeneity was observed (Figure 3).

We also calculated the summary positive likelihood ratio (PLR), negative likelihood ratio (NLR), diagnostic odds ratio (DOR), and the area under the SROC for each group (Table 2). The PLR in FNA group was 7.87, indicating that patients with PC had more than a 7.8-fold higher chance of a positive* K-ras* mutation in FNA sample compared to patients without PC. The NLR in ERCP was 0.34, which indicated that if the* K-ras* mutation in ERCP sample was negative, the probability of these patients developing PC was approximately 34%. Thus ERCP-negative results may not be used to exclude PC.

**Figure 4:**
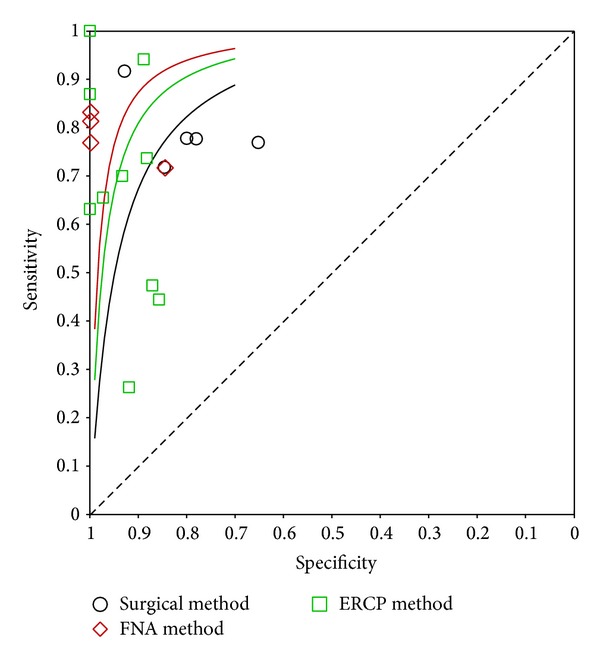
Summary receiver-operating characteristic curves for detecting* K-ras* mutation using FNA/ERCP/surgical methods from the hierarchical summary receiver operating characteristic model.

The SROC approach is the standard method in the meta-analysis of diagnostic reporting pairs of sensitivity and specificity [30]. This approach uses DOR as the main outcome measure which removes the effect of a possible threshold [31]. Therefore, we included the SROC curves obtained using the parameters of the hierarchical model to obtain an overall summary of moderately invasive methods and surgery. As shown in Figure 4, the SROC curve of paired data of moderately invasive methods and surgery indicated that the former curve falls above the surgery curve. Thus, it seems reasonable to conclude that* K-ras* mutation in cytohistological material obtained from ERCP or EUS-FNA may have better diagnostic value than that obtained from surgery. The difference may be caused by the different assays used to detect* K-ras* mutation. The materials obtained from ERCP or FNA usually have higher tissue specificity and can be sent for analysis immediately, while the intraoperative measurements of* K-ras* mutations in resection margins are far too complex. However, due to the limited number of studies in FNA and surgery group, this difference may have occurred by chance.

### 3.3. Diagnostic Accuracy of Serum* K-ras* Mutation Level for PC

The diagnostic value of serum* K-ras* mutation for detecting PC was reported in two articles [18, 22] in the same patient population, from which we sort out three individual studies. As shown in Table 3,* K-ras* mutation detection in cytohistological material had better value for early diagnosis due to its high sensitivity. However, detection of plasma* K-ras* mutations shows a high specificity, which may become a confirmatory tool in those cases in which a more invasive approach is contraindicated.

### 3.4. Summary Diagnostic Accuracy of* K-ras* Mutation versus Cytology for PC

**Figure 5:**
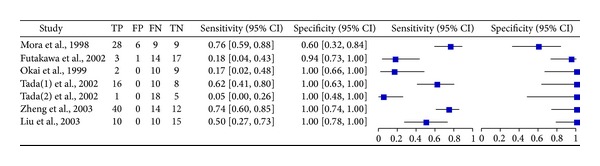
Forest plot of the meta-analysis of seven studies which included the diagnostic value of cytology for detecting PC.

Cancer is a diverse class of diseases that differ widely in their causes and biology; therefore, it is unlikely that a single marker will detect all cancers in a particular organ with high specificity and sensitivity. The diagnostic value of cytology for detecting PC was reported in seven studies, four of which also reported the diagnostic accuracy of* K-ras* in combination with cytopathology. The raw data for the two markers are shown in Figure 5. The pooled sensitivity and specificity for cytology were inferior to* K-ras* mutation detection: sensitivity 54% (95% CI: 47–61%); specificity 91% (95% CI: 83–96%); and DOR 7.52 (95% CI: 2.80–20.18). When two procedures were combined, the diagnostic accuracy was significantly improved with a pooled sensitivity of 83% (95% CI: 76–89%), specificity 100%, and DOR 79.46 (95% CI: 17.89–352.92).

### 3.5. Metaregression for Heterogeneity

The heterogeneity within the studies can also be observed in the SROC diagram. To investigate this heterogeneity, we attempted to explore the following study characteristics using metaregression: population characteristics (gender, ethnicity, age, disease types, and stage distribution), study design (prospective or retrospective and year of publication), and test characteristics (test type and number of tests per screening round). However, because of the unsatisfactory methodological quality of the studies, assay type and country were the only two features examined. The accuracy measure used was DOR, as it is a unitary measure of diagnostic performance that encompasses both sensitivity and specificity. However, due to the limited number of studies, the differences for assay type and country did not have a statistically significant effect on DOR (Table 4), suggesting that the influencing factors are complex.

### 3.6. Publication Bias

**Figure 6:**
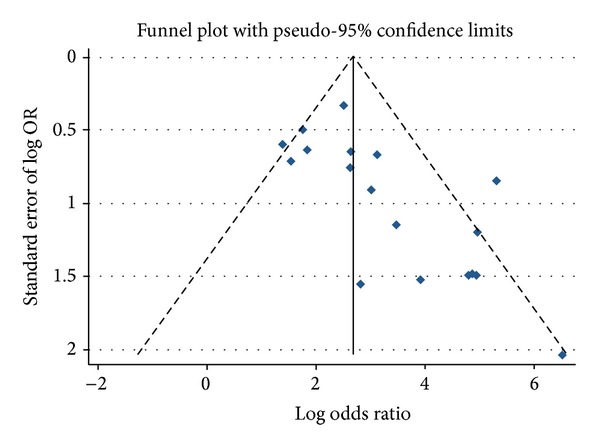
Deeks funnel plot output using the “metafunnel” command for Stata (version 12.0).

A Deeks funnel plot was obtained using the “metafunnel” command for Stata (version 12.0, StataCorp, College Station, TX, USA). As shown in Figure 6, the funnel plot was asymmetric, which indicated that publication bias existed in our study.

## 4. Discussion

We assessed the diagnostic accuracy of* K-ras* mutation for PC in 19 studies from 16 published articles. The results demonstrated that* K-ras* mutation in cytohistological tissue is a valuable molecular marker and an independent diagnostic tool for PC. Over half of the studies stated that the independent application of* K-ras* was favorable, while some other studies concluded that* K-ras* mutations could be useful in the diagnosis of PC when combined with cytohistopathology. Only a limited number of studies allowed us to evaluate the combined sensitivity and specificity of* K-ras* and other techniques; in these studies, we observed higher values of diagnostic sensitivity, which may have been the result of selective reporting bias. However, the studies showed numerous methodological limitations, a broad range of diagnostic accuracy values, and heterogeneity.

As has been concluded, the mean accuracy of* K-ras* detection is higher in cytohistological material than in serum.* K-ras* mutations can be detected in pancreatic tissue, pancreatic juice, bile, plasma, and stools. The sensitivity of* K-ras* detection in stool does not appear to be appropriate for diagnostic use. Plasma* K-ras* mutations can potentially be used as a screening method in asymptomatic patients, but these mutations have mainly been detected in patients with distant metastasis; thus, this marker is not useful for the early diagnosis of PC [32]. Collection of pancreatic juice during ERCP is technically easier than EUS-FNA. However, there was one drawback to the analysis of pancreatic juice. When there were even small cystic lesions in the pancreas, the mutant gene was also detected at very high frequency and amounts in these cases, which was comparable with cases of pancreatic neoplasms [33]. EUS-FNA has the advantage of targeting a solid mass lesion directly. On the other hand, pancreatic juice might reflect migrating tumor cells from a mass lesion, but actually cells with mutant* K-ras* gene from either malignant or benign cystic lesions are collected in it. Therefore, analysis of aspirates obtained by targeting under EUS should be superior to pancreatic juice.

Besides, different* K-ras* analysis systems are also shown in our meta-analysis. Several assays are available to detect* k-ras* mutation, such as PCR-ASO-DBH, PCR-RFLP, hybridization protection assay, PCR-SSCP, and PCR-denaturing gradient gel electrophoresis. Each of them has a different analytical sensitivity, which can be an important source of heterogeneity. Since these assays use different technologies, it is likely that there are differences in the accuracy between them. Watanabe et al. detected* K-ras* mutation in their study with a method (the mutant allele specific amplification (MASA) method) declared to be most sensitive for detecting point mutations for* K-ras* among various other methods [34]. Their results suggest that the detection of* K-ras* mutation can be achieved even in bile samples by using a highly sensitive method, such as MASA. Unfortunately, there is currently no standard test that has been approved by the US Food and Drug Administration in this regard.

Different incidence of* K-ras* mutations among distinct geographical areas is another reason to affect the diagnostic accuracy. In Japan, the prevalence of* K-ras* mutation ranged from 90 to 100%, whereas in the West it was relatively low, ranging from 65 to 70% in Europe and 85% in USA. In the present meta-analysis, the effects of this factor were not evaluated successfully because of lack of necessary data in the original studies.

The development of PC consists of multiple steps which include progression from intraepithelial proliferation with dysplasia to carcinoma in situ and finally to invasive cancer [35]. During the disease process, various genetic disorders occur which lead to loss of mechanisms that control cell differentiation, growth, and apoptosis [36]. These genetic alterations include point mutation in* K-ras*, overexpression of oncogenes (e.g., EGFR, c-erbB-2, c-erbB-3, and c-erbB-4), and inactivation of tumor suppressor genes (e.g., p16, p53, DPC4, and BRCA2) [35, 36].

Members of the epidermal growth factor receptor (EGFR) family are transmembrane receptors with tyrosine kinase activity and are overexpressed in 20% to 90% of ductal adenocarcinomas [37]. The TGF-b signalling pathway, which inhibits growth, is deregulated in a large proportion of pancreatic cancers and associated with underexpression of TGF-b receptors and mutation of the SMAD4/DCP4 gene [38]. p53, which functions in a variety of pathways to control the G1 and S phases of the cell cycle and to initiate DNA repair or apoptosis [39], is inactivated in at least 60% of pancreatic cancers. Many of the mutations result in production of a protein which has a greater stability and half-life than the wild type. p16, which binds to cdks 4 and 6 and prevents their association with the D-type cyclins, thus inhibiting the catalytic activity of the cdk-cyclin complex, is inactivated in over 80% of pancreatic cancers [40]. The relevance of these markers remains poorly defined. Most infiltrating pancreatic cancers accumulate numerous genetic alterations by the time they come to the clinical presentation. Probably* K-ras* mutations occur early and then the p16 gene expression alterations, while the inactivation of p53 and DPC4 genes appears late in pancreatic carcinogenesis. The detection of any of these genetic alterations could be useful for the early diagnosis of PC and for the differentiation between benign and malignant lesions.

More than 35 years after the discovery of the* K-ras* oncogene, the relevance of* K-ras* mutations in carcinogenesis is well established [41, 42]. The* K-ras* oncogene is activated by point mutations in 75–90% of pancreatic carcinoma. This gene belongs to the family of p21-ras genes that code for G-proteins, which are essential for intracellular signalling and thereby cellular proliferation. Mutations in the* K-ras* gene have been described at codons 12, 13, and 61. Because this mutation is commonly restricted to the codon 12, it is regarded as a “signature” of pancreatic carcinoma. Mutations in codon 13 occur rarely and are probably associated with familial pancreatic adenocarcinoma.* K-ras* mutation is a highly prevalent molecular change that occurs in the early stage of the pathogenesis of pancreatic malignancy. An understanding of the expression and regulation of this molecule might provide clues to facilitate new diagnostic and therapeutic approaches. In general, studies on the prognostic implications of* K-ras* mutations point towards a worse prognosis, but the findings have been inconsistent. Previous research has demonstrated a relationship between* K-ras* mutation and the presence of lymph node metastases. Niedergethmann et al. have reported that para-aortic lymph nodes diagnosed for* K-ras* mutation were independent pancreatic cancer prognostic markers in multivariate analysis [43]. All of the patients with the* K-ras* mutation in lymph nodes had recurrence after surgery and a significantly poorer survival than those without mutated* K-ras*.* K-ras* is now also widely accepted as a predictor of poor response to antiepidermal growth factor receptor (EGFR) monoclonal antibodies in metastatic colorectal cancer and testing for* K-ras* mutation has been incorporated into treatment [44]. Whether the importance for specific effector pathways of* K-ras* is different in different tumor types or whether findings from other malignancies can be translated to pancreatic cancer will have to be addressed in the future.

One of the major goals of meta-analysis is to explore reasons for heterogeneity rather than computation of a single summary measure [45]. In our meta-analysis, we failed to find a reason for the heterogeneity observed. Statistical analysis of the sources of heterogeneity may have been hampered due to the following reasons. First, many studies on diagnostic accuracy lacked information on key elements of design and conduct. Without complete and accurate reporting, we cannot correctly identify potential sources of bias and variability. Second, few studies using direct comparisons were available. The small numbers in this analysis also made subgroup analysis less accurate. Third, the diagnostic meta-analysis was also threatened by publication bias. The investigation of publication bias in diagnostic tests is problematic.

## 5. Conclusions

In conclusion, our meta-analysis found that the detection of* K-ras* mutation in cytohistological tissue is a valuable molecular marker and independent diagnostic tool for PC due to its high sensitivity and specificity. The detection of* K-ras* mutations may improve the diagnosis and treatment of one of the most aggressive malignancies worldwide. More studies are needed to further elucidate the combined value of* K-ras* mutation detection and cytology.

## Figures and Tables

**Table 1:** Main characteristics of the studies included.

Study	Country	TP/FP/FN/TN	Number	Invasive	Assay type
Trümper et al., 1994 [14]	Germany	16/0/0/11	1	Moderate	PCR-SSCP + enriched PCR-RFLP
Watanabe et al., 1998 [15]	Japan	19/1/10/36	2	Moderate	PCR-HPA
		22/5/6/32	3	Very	PCR-RFLP
Mora et al., 1998 [16]	Spain	35/0/8/16	4	Minimal	Enriched RFLP-PCR + SSCP
Wenger et al., 1999 [17]	Germany	28/3/8/12	5	Very	PCR + hybridization
Castells et al., 1999 [18]	Spain	28/4/11/22	6	Minimal	PCR-RFLP + SSCP
Okai et al., 1999 [19]	Japan	14/2/5/15	7	Moderate	PCR-RFLP
Futakawa et al., 2000 [20]	Japan	8/4/10/24	8	Moderate	PCR-RFLP
Tada et al., 2002a [21]	Japan	20/0/6/8	9	Minimal	Enriched PCR and enzyme linked minisequence assay
		12/0/7/5	10	Moderate	
Dianxu et al., 2002 [22]	China	33/1/3/13	11	Very	PCR-RFLP
Tada et al., 2002b [23]	Japan	10/5/28/57	12	Moderate	PCR-PHFA
		18/8/20/54	13	Moderate	PCR-ELMA
Zheng et al., 2003 [24]	China	45/0/9/12	14	Minimal	PCR-RFLP
Liu et al., 2003 [25]	China	14/1/6/14	15	Moderate	PCR-SSCP
Talar-Wojnarowska et al., 2005 [26]	Poland	20/8/6/15	16	Very	PCR-RFLP
Shi et al., 2008 [27]	USA	16/1/1/8	17	Moderate	LigAmp strategy
Parker et al., 2011 [28]	Spain	94/20/27/71	18	Very	PCR-RFLP
Ogura et al., 2012 [29]	Japan	266/0/40/47	19	Moderate	PCR-direct sequence assay

TP: true positive; FP: false positive; FN: false negative; and TN: true negative.

∗According to how the material was obtained, the types of sample used for *K-ras* analysis were divided into three groups: fine needle aspiration (FNA) was considered minimally invasive, endoscopic retrograde cholangio-pancreatography (ERCP) moderately invasive, and surgery very invasive.

**Table 2:** Summary of the diagnostic accuracy of *K-ras* mutation level in different materials in the same patient group using MetaDisc (Version 1.4).

	FNA	ERCP	Surgery
SEN	79 (95% CI: 72–85%)	76% (95% CI: 72–79%)	80% (95% CI: 74–85%)
SPE	94 (95% CI: 84–98%)	92% (95% CI: 89–95%)	79% (95% CI: 73–85%)
PLR	7.87 (95% CI: 2.95–20.97)	6.83 (95% CI: 3.40–13.73)	3.64 (2.42–5.47)
NLR	0.24 (95% CI: 0.18–0.32)	0.34 (95% CI: 0.19–0.58)	0.26 (0.20–0.36)
DOR	34.26 (95% CI: 10.01–117.2)	23.92 (95% CI: 8.20–69.77)	14.88 (95% CI: 7.51–29.47)
AUC	0.6957	0.9541	0.8540

SEN: sensitivity; SPE: specificity; PLR: positive likelihood ratio; NLR: negative likelihood ratio; DOR: diagnostic odds ratio; and AUC: area under curve.

**Table 3:** Summary of the diagnostic accuracy of *K-ras* mutation level in different materials in the same patient group using MetaDisc (version 1.4).

Summary	Serum	Cytohistological material
Sensitivity	41% (95% CI: 33–50%)	77% (95% CI: 74–80%)
Specificity	95% (95% CI: 90–99%)	88% (95% CI: 85–91%)
DOR	17.36 (95% CI: 6.61–45.59)	20.26 (95% CI: 11.40–36.03)

**Table 4:** Metaregression of the effects of methodological characteristics on diagnostic accuracy.

Var.	Coeff.	Std. Err.	*P* value	DOR	95% CI
Assay type	0.212	0.8041	0.7954	1.24	0.22–6.94
Country	0.431	0.9861	0.6690	1.54	0.19–12.75
